# Supplementary material for: Representativeness of individual-level data in COVID-19 phone surveys: Findings from Sub-Saharan Africa
Source: PLoS One. 2021 Nov 17;16(11):e0258877. doi: 10.1371/journal.pone.0258877 (PMC8598049; doi:10.1371/journal.pone.0258877)
Supplement: S1 Table — Ethiopia and Malawi. Notes: No weights are used. † denotes a dichotomous variable Respondent identifies whether the individual was a HFPS respondent–set to 1 for all individuals under the Phone Respondents column. These variables originate from the pre-COVID-19 F2F survey in each country. (PDF) [file pone.0258877.s001.pdf]

S 1 Table. Descriptive Statistics. Ethiopia and Malawi

	Ethiopia				Malawi			
	All F2F Adults (N=17563)		Phone Respondents (N=3196)		All F2F Adults (N=8582)		Phone Respondents (N=1700)	
	Mean	*SE*	*Mean*	*SE*	*Mean*	*SE*	*Mean*	*SE*
Individual-Level Attributes								
Respondent †	0.18	0.003	1.00	0.000	0.20	0.004	1.00	0.000
Head †	0.39	0.004	0.83	0.007	0.37	0.005	0.79	0.010
Spouse of head †	0.25	0.003	0.10	0.005	0.26	0.005	0.16	0.009
Child of head †	0.26	0.003	0.06	0.004	0.25	0.005	0.03	0.004
Male †	0.47	0.004	0.62	0.009	0.48	0.005	0.63	0.012
15-24 †	0.34	0.004	0.13	0.006	0.40	0.005	0.12	0.008
25-49 †	0.50	0.004	0.67	0.008	0.45	0.005	0.66	0.012
50+ †	0.16	0.003	0.21	0.007	0.16	0.004	0.23	0.010
Married †	0.54	0.004	0.66	0.008	0.56	0.005	0.76	0.010
Literate †	0.61	0.004	0.76	0.008	0.78	0.004	0.87	0.008
No Degree †	0.65	0.004	0.49	0.009	0.63	0.005	0.48	0.012
Primary Education †	0.20	0.003	0.22	0.007	0.14	0.004	0.13	0.008
Secondary Education †	0.08	0.002	0.14	0.006	0.10	0.003	0.18	0.009
Certificate †	0.01	0.001	0.02	0.002	0.09	0.003	0.13	0.008
Post-Secondary Education †	0.06	0.002	0.14	0.006	0.04	0.002	0.08	0.006
Sick in last 2-4 weeks †	0.14	0.003	0.17	0.007	0.22	0.005	0.24	0.010
Chronically ill/Disabled †	0.11	0.002	0.12	0.006	0.09	0.003	0.11	0.008
Employed for a wage/salary †	0.14	0.003	0.29	0.008	0.11	0.003	0.25	0.011
Owner of a household enterprise†	0.11	0.002	0.22	0.007	0.18	0.004	0.34	0.011
Casual laborer †	0.03	0.001	0.06	0.004	0.39	0.005	0.35	0.012
Individual owns a mobile phone †	0.42	0.004	0.81	0.007	0.36	0.005	0.82	0.009
Household-Level Attributes								
Household Size	5.00	0.018	4.05	0.038	5.38	0.027	4.74	0.055
Consumption Quintile 1 (lowest) †	0.13	0.003	0.05	0.004	0.17	0.004	0.09	0.007
Consumption Quintile 2 †	0.15	0.003	0.10	0.005	0.17	0.004	0.14	0.009
Consumption Quintile 3 †	0.16	0.003	0.13	0.006	0.17	0.004	0.16	0.009
Consumption Quintile 4 †	0.21	0.003	0.22	0.007	0.22	0.004	0.23	0.010
Consumption Quintile 5 (highest) †	0.34	0.004	0.51	0.009	0.27	0.005	0.37	0.012
