# Supplementary material for: Representativeness of individual-level data in COVID-19 phone surveys: Findings from Sub-Saharan Africa
Source: PLoS One. 2021 Nov 17;16(11):e0258877. doi: 10.1371/journal.pone.0258877 (PMC8598049; doi:10.1371/journal.pone.0258877)
Supplement: S2 Table — Nigeria and Uganda. Notes: No weights are used. † denotes a dichotomous variable. Respondent identifies whether the individual was a HFPS respondent–set to 1 for all individuals under the Phone Respondents column. These variables originate from the pre-COVID-19 F2F survey in each country. (PDF) [file pone.0258877.s002.pdf]

S 2 Table. Descriptive Statistics. Nigeria and Uganda.

	Nigeria				Uganda			
	All F2F Adults (N=15230)		Phone Respondents (N=1910)		All F2F Adults (N=8763)		Phone Respondents (N=2128)	
	Mean	*SE*	*Mean*	*SE*	*Mean*	*SE*	*Mean*	*SE*
Individual-Level Attributes								
Respondent †	0.13	0.003	1.00	0.000	0.24	0.005	1.00	0.000
Head †	0.33	0.004	0.83	0.009	0.35	0.005	0.74	0.010
Spouse of head †	0.28	0.004	0.09	0.007	0.22	0.004	0.20	0.009
Child of head †	0.30	0.004	0.06	0.006	0.32	0.005	0.04	0.004
Male †	0.48	0.004	0.73	0.010	0.48	0.005	0.52	0.011
15-24 †	0.32	0.004	0.06	0.005	0.38	0.005	0.06	0.005
25-49 †	0.45	0.004	0.55	0.011	0.41	0.005	0.60	0.011
50+ †	0.23	0.003	0.39	0.011	0.22	0.004	0.34	0.010
Married †	0.55	0.004	0.74	0.010	0.50	0.005	0.73	0.010
Literate †	0.74	0.004	0.82	0.009	0.75	0.005	0.78	0.009
No Degree †	0.34	0.004	0.24	0.010	0.52	0.005	0.48	0.011
Primary Education †	0.18	0.003	0.21	0.009	0.36	0.005	0.37	0.010
Secondary Education †	0.36	0.004	0.33	0.011	0.02	0.001	0.02	0.003
Certificate †	0.07	0.002	0.11	0.007	0.04	0.002	0.05	0.005
Post-Secondary Education †	0.06	0.002	0.11	0.007	0.06	0.003	0.08	0.006
Sick in last 2-4 weeks †	0.27	0.004	0.34	0.011	0.28	0.005	0.38	0.011
Chronically ill/Disabled †	0.11	0.003	0.13	0.008	0.03	0.002	0.02	0.003
Employed for a wage/salary †	0.10	0.002	0.21	0.009	0.18	0.004	0.24	0.009
Owner of a household enterprise†	0.26	0.004	0.42	0.011	0.17	0.004	0.32	0.010
Casual laborer †								
Individual owns a mobile phone †	0.80	0.003	0.95	0.005	0.39	0.005	0.76	0.009
Household-Level Attributes								
Household Size	6.80	0.031	5.54	0.077	6.29	0.032	5.41	0.059
Consumption Quintile 1 (lowest) †	0.16	0.003	0.10	0.007	0.20	0.004	0.16	0.008
Consumption Quintile 2 †	0.17	0.003	0.13	0.008	0.20	0.004	0.19	0.008
Consumption Quintile 3 †	0.20	0.003	0.18	0.009	0.20	0.004	0.21	0.009
Consumption Quintile 4 †	0.21	0.003	0.22	0.009	0.21	0.004	0.22	0.009
Consumption Quintile 5 (highest) †	0.26	0.004	0.38	0.011	0.19	0.004	0.23	0.009
