# Supplementary material for: Representativeness of individual-level data in COVID-19 phone surveys: Findings from Sub-Saharan Africa
Source: PLoS One. 2021 Nov 17;16(11):e0258877. doi: 10.1371/journal.pone.0258877 (PMC8598049; doi:10.1371/journal.pone.0258877)
Supplement: S3 Table — Notes: † denotes dichotomous variables. Standard errors are reported in parentheses. ***/**/* denote statistical significance at the 1/5/10 percent level, respectively. For each country the sample is all F2F survey household members age 15 and older for the set of households that were successfully interviewed in round 1 of the phone survey. (PDF) [file pone.0258877.s003.pdf]

S 3 Table. Marginal Effects from Logit Regressions on mobile ownership in the sampled household baseline datasets

	Ethiopia		*Malawi*		*Nigeria*		*Uganda*	
Household Size	-0.003		-0.000		-0.001		-0.004	*
	0.002		0.002		0.001		0.002	
Head †	0.261	***	0.317	***	0.200	***	0.374	***
	0.016		0.023		0.021		0.024	
Spouse of head †	0.064	***	0.019		0.110	***	0.237	***
	0.021		0.029		0.023		0.028	
Child of head †	-0.024	*	0.003		0.035	**	0.054	**
	0.014		0.019		0.015		0.021	
Male †	0.083	***	-0.007		0.016		0.025	**
	0.010		0.014		0.011		0.012	
Ages 15-29 †	0.000		0.000		0.000		0.000	
	.		.		.		.	
Ages 30-49 †	0.030	***	0.113	***	0.044	***	0.121	***
	0.011		0.016		0.014		0.017	
Ages 50+ †	-0.094	***	0.027		0.017		0.040	**
	0.014		0.019		0.016		0.017	
Married †	0.076	***	0.096	***	-0.000		0.103	***
	0.015		0.021		0.020		0.017	
No degree †	0.000		0.000		0.000		0.000	
	.		.		.		.	
Primary †	0.164	***	0.090	***	0.068	***	0.123	***
	0.012		0.016		0.017		0.011	
Secondary †	0.305	***	0.288	***	0.127	***	0.143	***
	0.016		0.019		0.015		0.031	
Certificate †	0.318	***	0.140	***	0.164	***	0.151	***
	0.043		0.021		0.019		0.024	
Post-Secondary Degree †	0.391	***	0.363	***	0.149	***	0.171	***
	0.021		0.035		0.024		0.021	
Employed for a wage/salary †	0.122	***	0.123	***	0.055	**	0.059	***
	0.014		0.021		0.023		0.014	
Owner of a household enterprise †	0.108	***	0.128	***	0.040	***	0.102	***
	0.013		0.015		0.012		0.013	
Casual laborer †	-0.015		-0.037	***				
	0.026		0.013					
Consumption quintile 1 †	0.000		0.000		0.000		0.000	
	.		.		.		.	
Consumption quintile 2 †	0.048	**	0.029		0.037	**	0.072	***
	0.022		0.023		0.017		0.017	
Consumption quintile 3 †	0.063	***	0.079	***	0.042	**	0.065	***
	0.021		0.023		0.017		0.017	
Consumption quintile 4 †	0.094	***	0.066	***	0.032	*	0.099	***
	0.021		0.021		0.018		0.017	
Consumption quintile 5 †	0.133	***	0.139	***	0.038	*	0.101	***
	0.021		0.023		0.019		0.018	
Spatial Fixed Effects	Region x Urban		District		State		Subregion	
Number of Observations	8535		4959		6183		6647	
Pseudo R-squared	0.358094154		0.365876544		0.161495771		0.352956342	
