# Supplementary material for: Representativeness of individual-level data in COVID-19 phone surveys: Findings from Sub-Saharan Africa
Source: PLoS One. 2021 Nov 17;16(11):e0258877. doi: 10.1371/journal.pone.0258877 (PMC8598049; doi:10.1371/journal.pone.0258877)
Supplement: S6 Table — Note: Base row reports the nationally representative mean among all adults in the face-to-face survey. Rows other than the base row report the difference from the base and a p-value from a test of significance for that difference. (PDF) [file pone.0258877.s006.pdf]

S 6 Table. Nigeria: Tests of difference between face-to-face adults and phone respondents, by sex and age group.

	Comparison Group		*National*			*Males*			*Females*			*Ages 15-24*			*Ages 25-49*			*Ages 50+*		
Variable	Sample	Weight	Beta	p-value		Beta	p-value		Beta	p-value		Beta	p-value		Beta	p-value		Beta	p-value	
Female	Base, All F2F Adults	F2F HH Weight	0.515									0.522			0.529			0.477		
	Phone respondents	HFPS HH Weight	-0.263	(.000)	***							-0.116	(.012)	**	-0.278	(.000)	***	-0.198	(.000)	***
	Phone respondents	HFPS Individual Weight	-0.068	(.004)	***							-0.096	(.042)	**	-0.150	(.000)	***	-0.182	(.000)	***
Ages 15-24	Base, All F2F Adults	F2F HH Weight	0.313			0.353			0.359											
	Phone respondents	HFPS HH Weight	-0.255	(.000)	***	-0.256	(.000)	***	-0.185	(.000)	***									
	Phone respondents	HFPS Individual Weight	-0.100	(.000)	***	-0.141	(.000)	***	-0.093	(.003)	***									
Ages 25-49	Base, All F2F Adults	F2F HH Weight	0.469			0.467			0.488											
	Phone respondents	HFPS HH Weight	0.082	(.000)	***	0.203	(.000)	***	0.101	(.000)	***									
	Phone respondents	HFPS Individual Weight	-0.010	(.622)		0.073	(.002)	***	0.070	(.038)	**									
Ages 50+	Base, All F2F Adults	F2F HH Weight	0.218			0.180			0.153											
	Phone respondents	HFPS HH Weight	0.173	(.000)	***	0.053	(.001)	***	0.084	(.001)	***									
	Phone respondents	HFPS Individual Weight	0.111	(.000)	***	0.069	(.001)	***	0.023	(.331)										
Head	Base, All F2F Adults	F2F HH Weight	0.326			0.566			0.188			0.074			0.475			0.703		
	Phone respondents	HFPS HH Weight	0.507	(.000)	***	0.353	(.000)	***	0.502	(.000)	***	0.391	(.000)	***	0.411	(.000)	***	0.268	(.000)	***
	Phone respondents	HFPS Individual Weight	0.120	(.000)	***	0.170	(.000)	***	0.127	(.000)	***	0.061	(.003)	***	0.170	(.000)	***	0.181	(.000)	***
Spouse	Base, All F2F Adults	F2F HH Weight	0.290			0.024			0.477			0.118			0.387			0.190		
	Phone respondents	HFPS HH Weight	-0.211	(.000)	***	-0.018	(.000)	***	-0.254	(.000)	***	-0.019	(.436)		-0.311	(.000)	***	-0.168	(.000)	***
	Phone respondents	HFPS Individual Weight	-0.049	(.034)	**	-0.001	(.881)		-0.006	(.860)		0.037	(.299)		-0.147	(.000)	***	-0.092	(.002)	***
Married	Base, All F2F Adults	F2F HH Weight	0.561			0.554			0.545			0.171			0.786			0.680		
	Phone respondents	HFPS HH Weight	0.175	(.000)	***	0.297	(.000)	***	-0.152	(.000)	***	0.110	(.003)	***	0.020	(.189)		0.046	(.100)	*
	Phone respondents	HFPS Individual Weight	0.050	(.034)	**	0.172	(.000)	***	0.016	(.610)		0.052	(.155)		0.013	(.478)		0.116	(.000)	***
Literate	Base, All F2F Adults	F2F HH Weight	0.751			0.623			0.424			0.709			0.480			0.231		
	Phone respondents	HFPS HH Weight	0.030	(.026)	**	0.002	(.935)		0.038	(.175)		0.158	(.000)	***	0.143	(.000)	***	0.089	(.002)	***
	Phone respondents	HFPS Individual Weight	0.021	(.253)		0.001	(.960)		0.057	(.115)		0.139	(.001)	***	0.092	(.000)	***	0.049	(.116)	
No degree	Base, All F2F Adults	F2F HH Weight	0.355			0.732			0.802			0.689			0.768			0.937		
	Phone respondents	HFPS HH Weight	-0.024	(.076)	*	-0.016	(.381)		-0.061	(.004)	***	-0.235	(.000)	***	-0.061	(.000)	***	-0.034	(.005)	***
	Phone respondents	HFPS Individual Weight	-0.025	(.218)		-0.025	(.296)		-0.071	(.014)	**	-0.201	(.000)	***	-0.044	(.046)	**	-0.005	(.676)	
Wage employment	Base, All F2F Adults	F2F HH Weight	0.100			0.124			0.059			0.060			0.124			0.059		
	Phone respondents	HFPS HH Weight	0.084	(.000)	***	0.024	(.032)	**	0.059	(.000)	***	0.085	(.000)	***	0.041	(.001)	***	0.011	(.394)	
	Phone respondents	HFPS Individual Weight	0.032	(.011)	**	-0.002	(.848)		0.037	(.022)	**	0.054	(.029)	**	0.013	(.314)		-0.010	(.401)	
Enterprise owner	Base, All F2F Adults	F2F HH Weight	0.281			0.103			0.092			0.049			0.144			0.067		
	Phone respondents	HFPS HH Weight	0.179	(.000)	***	0.056	(.000)	***	0.127	(.000)	***	0.147	(.000)	***	0.064	(.000)	***	0.011	(.430)	
	Phone respondents	HFPS Individual Weight	0.070	(.001)	***	0.017	(.181)		0.067	(.004)	***	0.061	(.025)	**	0.029	(.059)	*	-0.004	(.785)	
Mobile owner	Base, All F2F Adults	F2F HH Weight	0.797			0.402			0.218			0.229			0.396			0.217		
	Phone respondents	HFPS HH Weight	0.133	(.000)	***	0.313	(.000)	***	0.289	(.000)	***	0.417	(.000)	***	0.329	(.000)	***	0.261	(.000)	***
	Phone respondents	HFPS Individual Weight	0.060	(.001)	***	0.213	(.000)	***	0.177	(.000)	***	0.310	(.000)	***	0.207	(.000)	***	0.143	(.000)	***
