# Supplementary material for: Representativeness of individual-level data in COVID-19 phone surveys: Findings from Sub-Saharan Africa
Source: PLoS One. 2021 Nov 17;16(11):e0258877. doi: 10.1371/journal.pone.0258877 (PMC8598049; doi:10.1371/journal.pone.0258877)
Supplement: S7 Table — Notes: Base row reports the nationally representative mean among all adults in the face-to-face survey. Rows other than the base row report the difference from the base and a p-value from a test of significance for that difference. (PDF) [file pone.0258877.s007.pdf]

**S 7 Table. Uganda: Tests of difference between face-to-face adults and phone respondents, by sex and age group.**

| *Comparison Group* | | | *National* | | | *Males* | | | *Females* | | | *Ages 15-24* | | | *Ages 25-49* | | | *Ages 50+* | | |
|---|---|---|---|---|---|---|---|---|---|---|---|---|---|---|---|---|---|---|---|---|
| Variable | Sample | Weight | Beta | p-value | | Beta | p-value | | Beta | p-value | | Beta | p-value | | Beta | p-value | | Beta | p-value | |
| | Base, All F2F Adults | F2F HH Weight | 0.518 | | | | | | | | | 0.522 | | | 0.529 | | | 0.477 | | |
| Female | Phone respondents | HFPS HH Weight | -0.036 | (.020) | ** | | | | | | | -0.116 | (.012) | ** | -0.278 | (.000) | *** | -0.198 | (.000) | *** |
| | Phone respondents | HFPS Individual Weight | 0.039 | (.028) | ** | | | | | | | -0.096 | (.042) | ** | -0.150 | (.000) | *** | -0.182 | (.000) | *** |
| | Base, All F2F Adults | F2F HH Weight | 0.360 | | | 0.353 | | | 0.359 | | | | | | | | | | | |
| Ages 15-24 | Phone respondents | HFPS HH Weight | -0.290 | (.000) | *** | -0.256 | (.000) | *** | -0.185 | (.000) | *** | | | | | | | | | |
| | Phone respondents | HFPS Individual Weight | -0.205 | (.000) | *** | -0.141 | (.000) | *** | -0.093 | (.003) | *** | | | | | | | | | |
| | Base, All F2F Adults | F2F HH Weight | 0.450 | | | 0.467 | | | 0.488 | | | | | | | | | | | |
| Ages 25-49 | Phone respondents | HFPS HH Weight | 0.183 | (.000) | *** | 0.203 | (.000) | *** | 0.101 | (.000) | *** | | | | | | | | | |
| | Phone respondents | HFPS Individual Weight | 0.152 | (.000) | *** | 0.073 | (.002) | *** | 0.070 | (.038) | ** | | | | | | | | | |
| | Base, All F2F Adults | F2F HH Weight | 0.190 | | | 0.180 | | | 0.153 | | | | | | | | | | | |
| Ages 50+ | Phone respondents | HFPS HH Weight | 0.107 | (.000) | *** | 0.053 | (.001) | *** | 0.084 | (.001) | *** | | | | | | | | | |
| | Phone respondents | HFPS Individual Weight | 0.053 | (.000) | *** | 0.069 | (.001) | *** | 0.023 | (.331) | | | | | | | | | | |
| | Base, All F2F Adults | F2F HH Weight | 0.374 | | | 0.566 | | | 0.188 | | | 0.074 | | | 0.475 | | | 0.703 | | |
| Head | Phone respondents | HFPS HH Weight | 0.369 | (.000) | *** | 0.353 | (.000) | *** | 0.502 | (.000) | *** | 0.391 | (.000) | *** | 0.411 | (.000) | *** | 0.268 | (.000) | *** |
| | Phone respondents | HFPS Individual Weight | 0.072 | (.000) | *** | 0.170 | (.000) | *** | 0.127 | (.000) | *** | 0.061 | (.003) | *** | 0.170 | (.000) | *** | 0.181 | (.000) | *** |
| | Base, All F2F Adults | F2F HH Weight | 0.238 | | | 0.024 | | | 0.477 | | | 0.118 | | | 0.387 | | | 0.190 | | |
| Spouse | Phone respondents | HFPS HH Weight | -0.032 | (.012) | ** | -0.018 | (.000) | *** | -0.254 | (.000) | *** | -0.019 | (.436) | | -0.311 | (.000) | *** | -0.168 | (.000) | *** |
| | Phone respondents | HFPS Individual Weight | 0.110 | (.000) | *** | -0.001 | (.881) | | -0.006 | (.860) | | 0.037 | (.299) | | -0.147 | (.000) | *** | -0.092 | (.002) | *** |
| | Base, All F2F Adults | F2F HH Weight | 0.525 | | | 0.554 | | | 0.545 | | | 0.171 | | | 0.786 | | | 0.680 | | |
| Married | Phone respondents | HFPS HH Weight | 0.201 | (.000) | *** | 0.297 | (.000) | *** | -0.152 | (.000) | *** | 0.110 | (.003) | *** | 0.020 | (.189) | | 0.046 | (.100) | * |
| | Phone respondents | HFPS Individual Weight | 0.166 | (.000) | *** | 0.172 | (.000) | *** | 0.016 | (.610) | | 0.052 | (.155) | | 0.013 | (.478) | | 0.116 | (.000) | *** |
| | Base, All F2F Adults | F2F HH Weight | 0.795 | | | 0.623 | | | 0.424 | | | 0.709 | | | 0.480 | | | 0.231 | | |
| Literate | Phone respondents | HFPS HH Weight | 0.007 | (.488) | | 0.002 | (.935) | | 0.038 | (.175) | | 0.158 | (.000) | *** | 0.143 | (.000) | *** | 0.089 | (.002) | *** |
| | Phone respondents | HFPS Individual Weight | -0.018 | (.203) | | 0.001 | (.960) | | 0.057 | (.115) | | 0.139 | (.001) | *** | 0.092 | (.000) | *** | 0.049 | (.116) | |
| | Base, All F2F Adults | F2F HH Weight | 0.465 | | | 0.732 | | | 0.802 | | | 0.689 | | | 0.768 | | | 0.937 | | |
| No degree | Phone respondents | HFPS HH Weight | -0.029 | (.022) | ** | -0.016 | (.381) | | -0.061 | (.004) | *** | -0.235 | (.000) | *** | -0.061 | (.000) | *** | -0.034 | (.005) | *** |
| | Phone respondents | HFPS Individual Weight | 0.010 | (.544) | | -0.025 | (.296) | | -0.071 | (.014) | ** | -0.201 | (.000) | *** | -0.044 | (.046) | ** | -0.005 | (.676) | |
| | Base, All F2F Adults | F2F HH Weight | 0.219 | | | 0.124 | | | 0.059 | | | 0.060 | | | 0.124 | | | 0.059 | | |
| Wage employment | Phone respondents | HFPS HH Weight | 0.076 | (.000) | *** | 0.024 | (.032) | ** | 0.059 | (.000) | *** | 0.085 | (.000) | *** | 0.041 | (.001) | *** | 0.011 | (.394) | |
| | Phone respondents | HFPS Individual Weight | 0.035 | (.008) | *** | -0.002 | (.848) | | 0.037 | (.022) | ** | 0.054 | (.029) | ** | 0.013 | (.314) | | -0.010 | (.401) | |
| | Base, All F2F Adults | F2F HH Weight | 0.185 | | | 0.103 | | | 0.092 | | | 0.049 | | | 0.144 | | | 0.067 | | |
| Enterprise owner | Phone respondents | HFPS HH Weight | 0.135 | (.000) | *** | 0.056 | (.000) | *** | 0.127 | (.000) | *** | 0.147 | (.000) | *** | 0.064 | (.000) | *** | 0.011 | (.430) | |
| | Phone respondents | HFPS Individual Weight | 0.059 | (.000) | *** | 0.017 | (.181) | | 0.067 | (.004) | *** | 0.061 | (.025) | ** | 0.029 | (.059) | * | -0.004 | (.785) | |
| | Base, All F2F Adults | F2F HH Weight | 0.445 | | | 0.402 | | | 0.218 | | | 0.229 | | | 0.396 | | | 0.217 | | |
| Mobile owner | Phone respondents | HFPS HH Weight | 0.345 | (.000) | *** | 0.313 | (.000) | *** | 0.289 | (.000) | *** | 0.417 | (.000) | *** | 0.329 | (.000) | *** | 0.261 | (.000) | *** |
| | Phone respondents | HFPS Individual Weight | 0.185 | (.000) | *** | 0.213 | (.000) | *** | 0.177 | (.000) | *** | 0.310 | (.000) | *** | 0.207 | (.000) | *** | 0.143 | (.000) | *** |
