# Supplementary material for: Representativeness of individual-level data in COVID-19 phone surveys: Findings from Sub-Saharan Africa
Source: PLoS One. 2021 Nov 17;16(11):e0258877. doi: 10.1371/journal.pone.0258877 (PMC8598049; doi:10.1371/journal.pone.0258877)
Supplement: S8 Table — Notes: Base row reports the HFPS-based nationally representative mean among all adults present in the face-to-face and phone surveys. Rows other than the base row report the difference from the base and a p-value from a test of significance for that difference. Employment = 1 if individual spent any time in the last seven days doing specified work, 0 otherwise. All data are from the fifth round of the HFPS in Malawi and Nigeria. (PDF) [file pone.0258877.s008.pdf]

S 8 Table. Difference between adults and phone respondent employment outcomes, by sex.

				**Malawi**			**Nigeria**		
Variable	*Sample*	*Weight*	*Abbrev.*	*Beta*	*p-value*		*Beta*	*p-value*	
Any Employment	Adult Females (base)	HFPS HH Weight	w1	0.556			0.655		
	Female Respondents	HFPS HH Weight	w1	0.151	(.000)	***	0.07	(.007)	***
	Female Respondents	HFPS Individual Weight	w2	0.108	(.003)	***	0.035	(.290)	
Wage Employment	Adult Females (base)	HFPS HH Weight	w1	0.095			0.045		
	Female Respondents	HFPS HH Weight	w1	0.074	(.000)	***	0.03	(.061)	*
	Female Respondents	HFPS Individual Weight	w2	0.039	(.044)	**	0.024	(.202)	
Self-Employment	Adult Females (base)	HFPS HH Weight	w1	0.107			0.328		
	Female Respondents	HFPS HH Weight	w1	0.071	(.000)	***	0.007	(.805)	
	Female Respondents	HFPS Individual Weight	w2	0.076	(.004)	***	-0.021	(.509)	
Any Employment	Adult Males (base)	HFPS HH Weight	w1	0.667			0.788		
	Male Respondents	HFPS HH Weight	w1	0.22	(.000)	***	0.093	(.000)	***
	Male Respondents	HFPS Individual Weight	w2	0.201	(.000)	***	0.052	(.018)	**
Wage Employment	Adult Males (base)	HFPS HH Weight	w1	0.219			0.128		
	Male Respondents	HFPS HH Weight	w1	0.039	(.022)	**	-0.004	(.719)	
	Male Respondents	HFPS Individual Weight	w2	0.024	(.345)		0.004	(.812)	
Self-Employment	Adult Males (base)	HFPS HH Weight	w1	0.13			0.264		
	Male Respondents	HFPS HH Weight	w1	0.091	(.000)	***	0.051	(.000)	***
	Male Respondents	HFPS Individual Weight	w2	0.117	(.000)	***	0.022	(.320)	
