# Supplementary material for: Representativeness of individual-level data in COVID-19 phone surveys: Findings from Sub-Saharan Africa
Source: PLoS One. 2021 Nov 17;16(11):e0258877. doi: 10.1371/journal.pone.0258877 (PMC8598049; doi:10.1371/journal.pone.0258877)
Supplement: S9 Table — Notes: Base row reports the HFPS-based nationally representative mean among all adults present in the face-to-face and phone surveys. Rows other than the base row report the difference from the base and a p-value from a test of significance for that difference. Employment = 1 if individual spent any time in the last seven days doing specified work, 0 otherwise. All data are from the fifth round of the HFPS in Malawi and Nigeria. (PDF) [file pone.0258877.s009.pdf]

S 9 Table. Difference between adults and phone respondent employment outcomes, by age group.

Variable	*Sample*	*Weight*	*Abbrev.*	Malawi *Beta*	Malawi *p-value*		Nigeria *Beta*	Nigeria *p-value*	
Any Employment	Adults Ages 15-24 (base)	HFPS HH Weight	w1	0.376			0.597		
	Respondents Ages 15-24	HFPS HH Weight	w1	0.387	(.000)	***	0.149	(.002)	***
	Respondents Ages 15-24	HFPS Individual Weight	w2	0.352	(.000)	***	0.06	(.292)	
Wage Employment	Adults Ages 15-24 (base)	HFPS HH Weight	w1	0.094			0.097		
	Respondents Ages 15-24	HFPS HH Weight	w1	0.141	(.001)	***	0.031	(.485)	
	Respondents Ages 15-24	HFPS Individual Weight	w2	0.08	(.096)	*	-0.002	(.956)	
Self-Employment	Adults Ages 15-24 (base)	HFPS HH Weight	w1	0.024			0.167		
	Respondents Ages 15-24	HFPS HH Weight	w1	0.166	(.001)	***	0.106	(.070)	*
	Respondents Ages 15-24	HFPS Individual Weight	w2	0.197	(.001)	***	0.061	(.222)	
Any Employment	Adults Ages 25-49 (base)	HFPS HH Weight	w1	0.751			0.772		
	Respondents Ages 25-49	HFPS HH Weight	w1	0.074	(.000)	***	0.096	(.000)	***
	Respondents Ages 25-49	HFPS Individual Weight	w2	0.005	(.838)		0.063	(.011)	**
Wage Employment	Adults Ages 25-49 (base)	HFPS HH Weight	w1	0.212			0.086		
	Respondents Ages 25-49	HFPS HH Weight	w1	0.039	(.004)	***	0.048	(.000)	***
	Respondents Ages 25-49	HFPS Individual Weight	w2	0.006	(.738)		0.034	(.055)	*
Self-Employment	Adults Ages 25-49 (base)	HFPS HH Weight	w1	0.188			0.387		
	Respondents Ages 25-49	HFPS HH Weight	w1	0.046	(.000)	***	-0.014	(.441)	
	Respondents Ages 25-49	HFPS Individual Weight	w2	0.053	(.026)	**	-0.019	(.470)	
Any Employment	Adults Ages 50+ (base)	HFPS HH Weight	w1	0.75			0.772		
	Respondents Ages 50+	HFPS HH Weight	w1	0.057	(.009)	***	0.038	(.010)	***
	Respondents Ages 50+	HFPS Individual Weight	w2	0.035	(.398)		-0.024	(.537)	
Wage Employment	Adults Ages 50+ (base)	HFPS HH Weight	w1	0.141			0.067		
	Respondents Ages 50+	HFPS HH Weight	w1	0.007	(.679)		0.005	(.490)	
	Respondents Ages 50+	HFPS Individual Weight	w2	-0.03	(.073)	*	0.009	(.662)	
Self-Employment	Adults Ages 50+ (base)	HFPS HH Weight	w1	0.131			0.26		
	Respondents Ages 50+	HFPS HH Weight	w1	-0.001	(.933)		-0.013	(.418)	
	Respondents Ages 50+	HFPS Individual Weight	w2	0.007	(.830)		-0.026	(.350)	
